# Supplementary material for: Social knowledge about others is anchored to self-knowledge in the hippocampal formation
Source: PLoS Biol. 2025 Apr 2;23(4):e3003050. doi: 10.1371/journal.pbio.3003050 (PMC11964405; doi:10.1371/journal.pbio.3003050)
Supplement: S1 Text — Fig A in S1 Text. Related to Fig 2. Significant quadratic relationship between accuracy and RDgroup (t(19) = 3.39, p = 0.0015).The underlying data for Fig A in S1 Text can be found in DOI: https://doi.org/10.6084/m9.figshare.28295732.v2 under “Data”. Fig B in S1 Text. Whole-brain searchlight analysis related to Fig 3. (A) Whole-brain group effect of rating discrepancy group (RDgroup) centered on left ventrolateral prefrontal cortex peak (x = −54; y = 20; z = 2; pFWE = .039). (B) Whole-brain group effect of group rating consistency (RCgroup) centered on the left hippocampus peak (x = −16; y = −34; z = −4; pFWE = .038). For visualization purposes, the images are displayed at an uncorrected statistical threshold of p < 0.005. The underlying data for Fig B in S1 Text can be found in DOI: https://doi.org/10.6084/m9.figshare.28295732.v2. Fig C in S1 Text. Whole-brain searchlight analysis for model matrices related to Fig 4. (A) Left Thalamus peak (x = −7, y = −16, z = 9, pFWE < 0.001). (B) Ventral striatum peak (x = 27, y = 18, z = 6). For visualization purposes, the images are displayed at an uncorrected statistical threshold of p < 0.001.The underlying data for Fig C in S1 Text can be found in DOI: https://doi.org/10.6084/m9.figshare.28295732.v2. Fig D in S1 Text. Explanation of Model-RDM Calculations. Related to Fig 4. (A) Example trial types during transformation, each coded with specific color, involving a particular individual and group. (B) Entries of the matrix, where each cell of the matrix represents a comparison between two trials. (C) Each row represents a distinct model, while each column represents which pair of trials is coded as similar or dissimilar based on the model criteria. For the individual model, trials involving the same individual are considered similar, while trials involving different individuals are considered dissimilar. In the group model, trials involving the same group are computed as similar, while those involving different groups are dis [file pbio.3003050.s001.pdf]

Supporting information

Multi-echo data fMRI pre-processing

This study used a multiband multi-echo (MBME) scanning sequence. We used TEDANA to combine the images[58-61]. Before images were combined, preprocessing was performed using fMRIPrep 22.1.1[62]; RRID:SCR_016216), which is based on Nipype 1.8.5([50], RRID:SCR_002502). Outputs of individual echoes after slice-timing correction, head-motion correction, and susceptibility distortion correction were introduced into the TEDANA pipeline to denoise data and combine the images. To normalize scanner-space TEDANA denoised data into MNI space, we used ANTS's antsApplyTransforms tool. For more details on the fMRIprep pipeline see https://fmriprep.readthedocs.io/en/stable/workflows.html.

<u>105 Everyday Scenarios</u>

1.Waking up early during the week
2.Taking an online class
3. Ironing clothes
4. Uploading picture to your social network
5. Playing board games
6. Devoting time to choose everyday clothing
7. Decorating their place
8. Having good sleep habits
9. Taking a dance class
10. Owning a pair of running shoes
11. Visiting a library
12. Talking on the phone
13. Talking on the phone in a foreign language
14. Using a motorbike
15. Buying clothes from a second-hand shop
16. Going to bed late
17. Going to a nightclub
18. Reading a novel
19. Taking a nap
20. Having a meeting with a co-worker of a different nationality
21. Playing football with your friends
22. Having a second house in the same province/state
23. Ordering take away pizza
24. Communicate via email
25. Listening to commercial music
26. Cycling bike
27. Sleeping more than 7 hours a night
28. Going out to dinner with friends.
29. Drinking coffee in the morning
30. Watching a romantic movie
31. Eating sushi
32. Speaking on the phone in a foreign language
33. Riding the bus
34. Watching cartoons

35. Following politics
36. Watching TV on evening
37. Drinking tea
38. Falling asleep in the car
39. Playing padel
40. Eating brunch
41. Reading newspaper
42. Playing video games
43. Eating a kebab
44. Following fashion trends
45. Visiting family
46. Throwing a house party
47. Listening to music in a foreign language
48. Fixing things around the house
49. Chat via instant messaging
50. Eating vegetables
51. Eating paella on Sunday
52. Eating fast food
53. Downloading a movie
54. Going to a concert
55. Painting their home
56. Meeting friends at the pub
57. Wearing a wool sweater
58. Eating spicy food
59. Smoking a cigarette
60. Speaking Valencian with friends and family
61. Growing vegetables
62. Wearing a leather jacket
63. Going to a work conference
64. Eating exotic meal
65. Knowing your friend’s grandparents
66. Sending a message using mostly emojis
67. Traveling abroad
68. Spending a weekend on the sofa
69. Going to a yoga class
70. Taking a coffee break at work
71. Listening to podcast
72. Spending a lot of money in clothes
73. To use laptop to take notes
74. Drinking milk
75. Wearing boots
76. Changing jobs
77. Falling asleep in front of the television
78. Holidaying in a tropical location
79. Personally knowing your neighbors
80. Greeting strangers when you enter in a public space
81. Using a dishwasher
82. Buying a new car

83. Watching sport on TV
84. Going to the beach during the week
85. Taking the train
86. Hiring a person to clean their house
87. Cooking dinner
88. Joining a political march
89. Watching a soap opera
90. Socializing with people older than you
91. Speak via video chat
92. Going for a hike
93. Attending a musical
94. Jogging 5 miles
95. Visiting an art museum
96. Wearing a hat
97. Driving to work
98. Meditating
99. Receiving a work-related phone call.
100. Taking more than an hour to get ready in the morning
101. Catching a taxi
102. Being accustomed to traffic
103. Living close to nature
104. Buying designer clothes
105. Doing sudoku

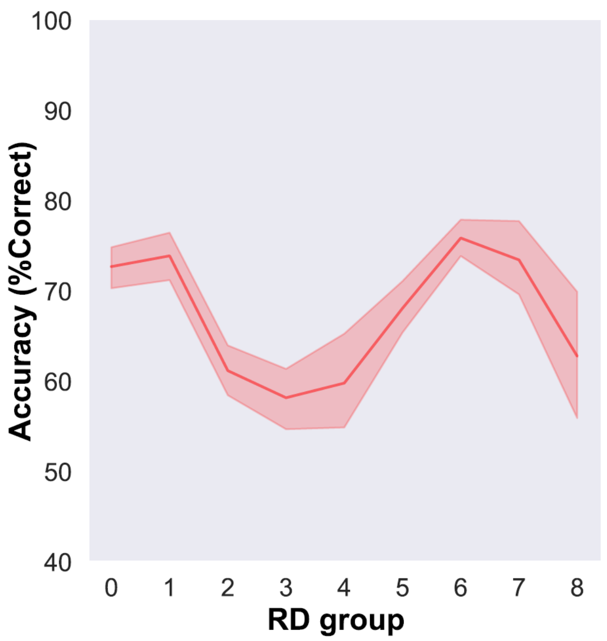

S1 Figure. Significant quadratic relationship between accuracy and RD_{group} ($t(19)=3.39$, $p=0.0015$).The underlying data for S1 Fig can be found in DOI:10.6084/m9.figshare.28295732 under “Data” .

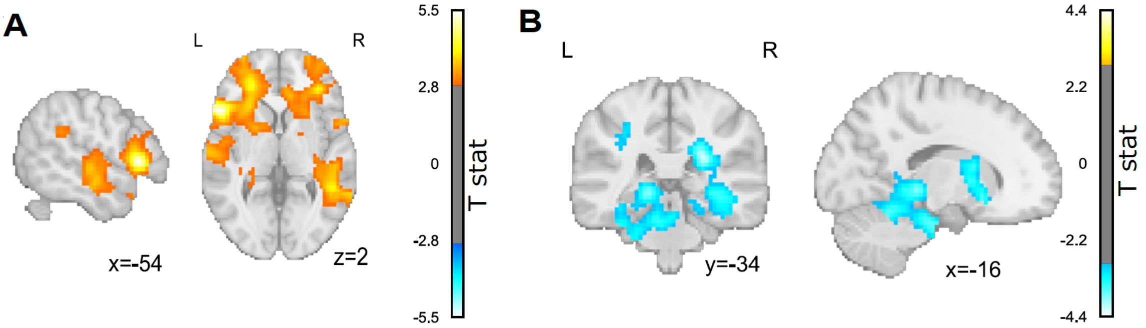

S2 Figure. Whole-brain searchlight analysis related to Fig.3. A. Whole-brain group effect of rating discrepancy group(RD_{group}) centered on left ventrolateral prefrontal cortex peak (x=-54;y=20;z=2; pFWE=.039). B. Whole-brain group effect of group rating consistency(RC_{group}) centered on the left hippocampus peak(x=-16;y=-34;z=-4;pFWE=.038). For visualization purposes, the images are displayed at an uncorrected statistical threshold of p<0.005. The underlying data for S2 Fig can be found in DOI: 10.6084/m9.figshare.28295732 .

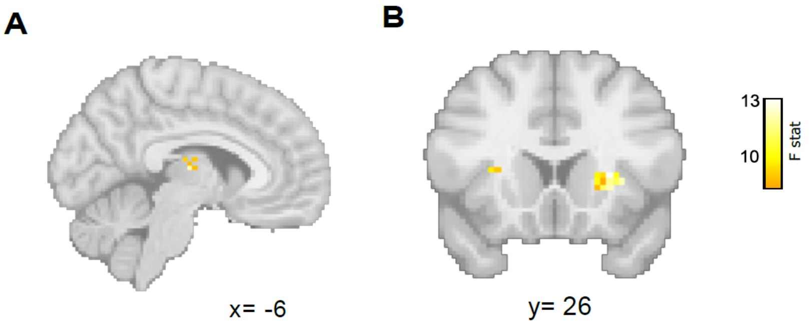

S3 Figure. Whole-brain searchlight analysis for model matrices related to Fig.4. A. Left Thalamus peak (x=-7,y= -16,z= 9, pFWE<0.001). B. Ventral striatum peak(x=27, y=18,z= 6). For visualization purposes, the images are displayed at an uncorrected statistical threshold of p<0.001.The underlying data for S3 Fig can be found in DOI:10.6084/m9.figshare.28295732 .

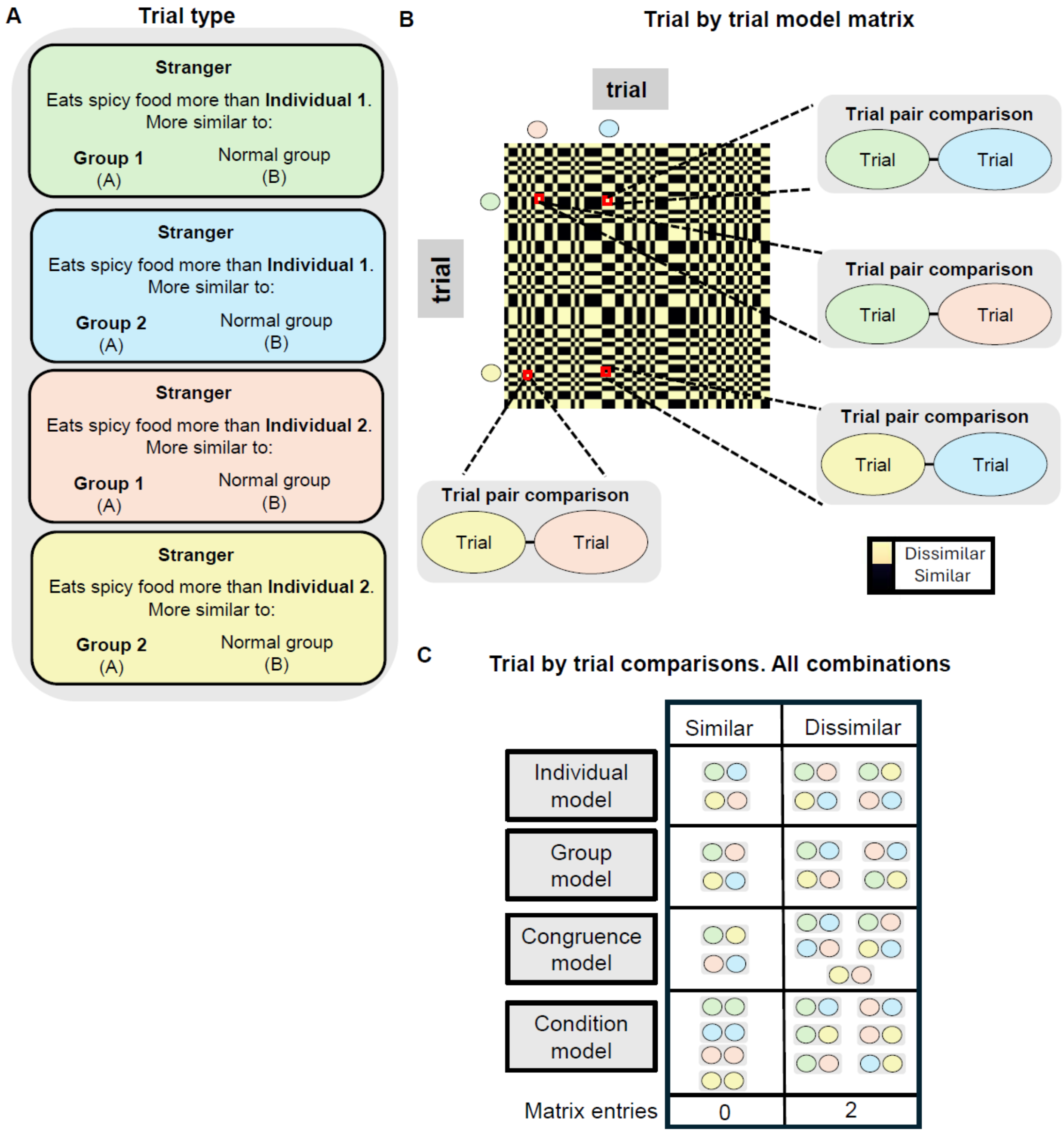

S4 Figure. Explanation of Model-RDM Calculations. Related to Fig. 4. A. Example trial types during transformation, each coded with specific color, involving a particular individual and group. B. Entries of the matrix, where each cell of the matrix represents a comparison between two trials. C. Each row represents a distinct model, while each column represents which pair of trials is coded as similar or dissimilar based on the model criteria. For the individual model, trials involving the same individual are considered similar, while trials involving different individuals are considered dissimilar. In the group model, trials involving the same group are computed as similar, while those involving different groups are dissimilar. For the congruence model, trials with the same congruence condition (e.g., individual 1-group 1 vs. individual 2-group 2) are assigned high similarity, while incongruent conditions are treated as dissimilar. Finally, in the condition model, only pairs of trials within the same condition are computed as similar, all other comparisons are dissimilar**.** For matrix entries, a 0 is assigned to comparisons with similar hypothesized representation and a 2 to those considered dissimilar across all models.

Region	x	y	z	Cluster Size	Peak Level T	Peak Level p(FWE-corr)
Left ventrolateral PFC	-54	20	2	133	5.55	0.039
Left entorhinal cortex	-18	-24	-22	30	4.37	0.015 *
Left hippocampus	-22	-18	-18	3	3.78	0.046 *

S1 Table. Brain areas that were significantly modulated by group rating discrepancy related to S2A Fig. * =small-volume corrected FWE for bilateral entorhinal cortex and bilateral hippocampus mask respectively.

Region	x	y	z	Cluster Size	Peak-level T	Peak-level p(FWE-corr)
Left entorhinal cortex	-18	-34	-6	3	3.68	0.021*
Left hippocampus	-16	-34	-4	2	3.83	0.038*
Right hippocampus	32	-30	-4	1	3.61	0.039*

S2 Table. Brain areas that were significantly modulated by differences in group rating consistency related to S2B Fig. *= cluster-level correction for bilateral entorhinal cortex and bilateral hippocampus mask respectively.

Region	x	y	z	Cluster Size	Cluster Level p(FWE-corr)	F	Peak Level p(FWE-corr)
Right ventral striatum	26	18	32	29	$< .002$	13.43	$< .001$
Left thalamus	-6	-16	8	28	$< .002$	12.38	$< .001$

S3 Table. Brain areas that were significantly modulated by the difference between the three models (group, individual, and congruence) related to S3 Fig.
